# The Tinetti Balance Test Is an Effective Predictor of Functional Decline in Non-Hospitalized Post-COVID-19 Individuals: A Cross-Sectional Study

**DOI:** 10.3390/jcm13216626

**Published:** 2024-11-04

**Authors:** Janice R. M. Bastos, Arthur S. Ferreira, Agnaldo J. Lopes, Talita P. Pinto, Erika Rodrigues, Fabio V. dos Anjos

**Affiliations:** 1 Rehabilitation Sciences Post-Graduation Program, Augusto Motta University (UNISUAM), Rio de Janeiro 21041-020, Brazil; 2 Physiotherapy Course, UNDB University Center, São Luís 65075-441, Brazil; 3 Instituto D’Or de Pesquisa e Ensino (IDOR), Rio de Janeiro 22281-100, Brazil; 4 Medical Sciences Post-Graduation Program, School of Medical Sciences, State University of Rio de Janeiro (UERJ), Rio de Janeiro 20550-013, Brazil

**Keywords:** COVID-19, postural control, functional status

## Abstract

**Background/Objectives:** Individuals with post-COVID-19 conditions risk developing short- and/or long-term neuromuscular impairments, including postural imbalance. However, there is limited evidence showing whether balance deficits are associated with declines in the functional status in post-COVID-19 individuals. This study examined postural balance in non-hospitalized post-COVID-19 individuals using different assessment tools and tested the most relevant balance tools in predicting functional status. **Methods:** This cross-sectional study enrolled 60 adults split into control (n = 30) and post-COVID-19 (n = 30) groups. Postural balance was assessed in both groups using the Functional Reach Test (FRT), Berg Balance Scale (BBS), Timed Up and Go (TUG), Tinetti Balance Test (Tinetti), and Mini-BESTest (MBT). Functional status in the post-COVID-19 group was assessed using post-COVID-19 functional status (PCFS). **Results:** Significant differences in postural stability between groups were found only for the FRT. All balance tests showed a statistically significant correlation with PCFS in the post-COVID-19 group, with better performance in all tests being associated with better functional status: Tinetti (r = −0.584), FRT (r = −0.542), MBT (r = −0.530), BBS (r = −0.415) and TUG (r = 0.368). Tinetti was the independent variable that significantly played an important role in determining PCFS (adjusted *R*^2^ = 0.318, *p* < 0.001). **Conclusions:** Post-COVID-19 functional status is best determined by the Tinetti Balance Test, making it an effective tool for assessing postural balance deficits in this population, with potential implications for postural control assessment and rehabilitation.

## 1. Introduction

The Severe Acute Respiratory Syndrome Coronavirus 2 (SARS-CoV-2) pandemic is one of the most significant in recent centuries, affecting millions worldwide [1]. COVID-19 is characterized as a multisystem disease due to its association with a variety of short- and long-term symptoms [2,3]. Some of the most common post-COVID-19 symptoms and complications, such as cardiovascular changes, pain, diffuse myalgia, fatigue, muscle weakness, and emotional changes (e.g., depression and anxiety [4,5]), can greatly affect the individual’s functional capacity. Therefore, assessment tools have been developed to evaluate the temporal course of the disease and its impact on the functional status of this population.

Klok and colleagues (2020) proposed a simple tool to measure and track the functional status of post-COVID-19 patients, called the post-COVID-19 functional status scale (PCFS) [6]. This scale was associated with various symptoms that affect the individual’s functional capacity during activities of daily living (ADL), such as fatigue, muscle weakness, pain, anxiety, and depression [5,7]. Because PCFS measures a full spectrum of functional outcomes, it has been considered as a primary endpoint in clinical trials involving COVID-19 infection. In this context, PCFS may serve as a crucial tool for assessing the impact of motor dysfunction potentially associated with post-COVID-19 syndrome and may help to identify different chronic manifestations caused by COVID-19.

Postural balance is a crucial aspect of motor function that appears to be impaired in the acute and post-acute phases of COVID-19. Biomechanically, postural balance involves the integration of sensorimotor strategies to control the centre of gravity within the support base (the feet in stance [8]). In both the acute and post-acute phases of COVID-19, patients were found to have poor postural balance compared to a control group, regardless of age [9,10,11,12]. These findings suggest that postural instability appears to be a functional outcome after COVID-19. However, there is limited evidence showing whether such balance changes reflect a decline in the functional status in post-COVID-19 individuals, and where correlations have been estimated, they have often been based on non-specific post-COVID-19 scales [13]. Therefore, the assessment of postural balance and its association with post-COVID-19 functional status seems to be of potential interest for better characterizing motor function rehabilitation in post-COVID-19 individuals. Moreover, whether a given accessible test can effectively predict post-COVID-19 functional status in clinical practice is still an open question.

Therefore, this study aimed to investigate whether postural balance is affected in non-hospitalized individuals with post-COVID-19 conditions using different assessment instruments. In addition, we investigated whether any changes in postural balance were correlated with the level of functionality as measured by the PCFS scale. Our hypothesis is that post-COVID-19 individuals show deficits in postural balance that may be associated with the degree of functional impairment measured by the PCFS scale. We also asked whether a given balance tool would best predict post-COVID-19 functional status. Given that a considerable proportion of non-hospitalized persons experience post-COVID-19 symptoms [2,14], a better understanding of the postural balance in this group can have potential implications for the assessment and rehabilitation of postural balance in post-COVID-19 individuals.

## 2. Materials and Methods

### 2.1. Study Design, Participants, and Ethical Aspects

This is a cross-sectional study with participants recruited from the Specialized Rehabilitation Center of Olho d’Água, CER III (São Luís, Maranhão, Brazil), between January and July 2022. The study was approved by the Research Ethics Committee of Augusto Motta University (CAAE–54483421.8.0000.5235; Rio de Janeiro, Brazil) and followed the principles of the Declaration of Helsinki. All participants signed the informed consent form before participating in the study.

Sixty (60) participants were split into two groups: a control group and a post-COVID-19 group (n= 30 each). This sample size was based on previous studies showing that a sample size of 18 to 43 individuals per group would have the power to detect differences between patients with COVID-19 and healthy subjects in dynamic balance performance, e.g., the Timed Up and Go (TUG) test and the Mini-BESTest (MBT) (α = 5%, β = 80%, [9,10]). Inclusion criteria for the post-COVID-19 group were a positive diagnosis for COVID-19 (RT-qPCR positive or serology), individuals who were not hospitalized, time since COVID-19 infection being at least one month [10], and admission to rehabilitation for sequelae. The control group consisted of patients selected for the physical and/or intellectual rehabilitation program without a previous diagnosis of COVID-19. All participants were over 18 years of age and had received the second dose of the vaccine. The evaluation of the control group was conducted prior to the start of the rehabilitation program. Exclusion criteria for both groups included neurological diseases, pre-existing musculoskeletal or neurological disorders, and pre-existing visual deficits that would affect postural control and walking.

### 2.2. Assessments

Initially, all participants were interviewed to collect sociodemographic information, anthropometric measures (body mass and height), and medical history (pre-existing comorbidities, current symptoms, and time since COVID-19-related infection). Previous comorbidities and current symptoms were self-reported.

#### 2.2.1. Postural Balance Assessment

For both groups, the classic tests for postural balance assessment were used: Functional Reach Test (FRT), Berg Balance Scale (BBS), TUG, Tinetti, and MBT. The choice of these tests was based on the instruments used to assess postural balance in COVID-19 patients [9,13] and because these tests are complementary, with different characteristics and limitations [15]. All tests were performed in the assessment room of the multiprofessional team at the Rehabilitation Centre by the same evaluator, using resources and verbal commands as previously described [15,16,17,18]. The duration of the assessment protocol ranged from 40 to 50 min, taking into account 5 min intervals between the application of each balance test.

The FRT was developed as a dynamic measure of balance and is used to assess individuals’ ability to stand and lean forward without losing balance [17,19]. A measuring tape was attached to the wall, parallel to the floor, and positioned at the level of the subject’s acromion. The participant was barefoot and positioned with feet hip-width apart, perpendicular to the wall, and close to the beginning of the measuring tape. With the wrists in a neutral position, elbows extended, and shoulders flexed at 90°, the volunteer was instructed to lean forward without touching the tape or moving their feet, and then the displacement over it was checked. The functional reach was obtained by averaging the distances reached over the three trials, with a measurement of 17 cm or less indicating a high risk of falling [15].

TUG aims to assess mobility [20,21] and was proposed to assess functional capacity in individuals following COVID-19 [22]. The test quantifies the time (in seconds) to stand up from a chair, walk 3 m, and return to the seated position [21]. The reference time for completing the test is up to 10 s, which is considered the normal time for healthy, independent adults with no risk of falling. Specifically, times between 11 and 20 s are expected for older persons with disabilities or frailty, partial independence, and low fall risk, while times greater than 20 s are expected for older persons with significant physical mobility deficits and a risk of falling [16].

The BBS assesses functional balance performance based on 14 items commonly encountered in daily life. The test is simple, easy to apply, and safe for assessing patients regardless of age [23]. Each item of the BBS consists of a five-point ordinal scale ranging from 0 to 4 points. The maximum score is 56 points, based on the time that a body position can be maintained, the distance the upper limb can reach in front of the body, and the time taken to complete the task. The lower the individual’s score, the greater their risk of falling. Specifically, scores below 46 points indicate a high risk of falling; scores above 53 points reflect a low risk of falling; and scores between 46 and 53 points indicate a moderate risk of falling [23,24].

The Tinetti test consists of several tasks that are representative of ADL and are scored by the examiner’s observation. This test is divided into two parts: one assessing balance and the other evaluating gait [25]. Scores on the Tinetti scale, which originally had 14 tasks (8 in the balance scale and 6 in the gait scale), range from 0 to a maximum of 28 points. Scores below 19 points and those between 19 and 24 points represent a high and moderate fall risk, respectively [18].

The MBT was developed as a shorter version of the Balance Evaluation Systems Test (BESTest). It is a clinical tool that assesses dynamic balance and is conducted in 10 to 15 min, containing items evenly distributed across four of the six sections of the original BESTest [26]. Its items are scored from 0 to 2, with a maximum score of 28 and a minimum score of 0, with higher scores indicating better postural balance [17].

#### 2.2.2. Assessment of Functional Status in Post-COVID-19

The PCFS scale was applied only to the post-COVID-19 group to determine the severity of their functional limitations. The PCFS scale was translated and cross-culturally adapted to Brazilian Portuguese for the functional assessment of non-hospitalized individuals with post-COVID-19 [27]. The scale was designed to cover the full range of functional limitations with six possible levels: from grade 0, “no functional limitations” to grade 4, “severe functional limitations”, and grade 5, “death” [6]. To appropriately assign the grade on the PCFS scale, which was answered by the patients themselves, the version of the questionnaire that assesses how COVID-19 affects their daily life was used, providing answers based on limitations. Then, a flowchart was presented to the individual, following the PCFS manual [6], which contains dichotomous yes/no questions, including inquiries ranging from living alone to the need to avoid or reduce tasks.

### 2.3. Statistical Analysis

A parametric approach was used based on the results of the Kolmogorov–Smirnov test. The Student’s *t*-test for independent samples was used to compare anthropometric data and balance test scores between groups (control and post-COVID-19). The Chi-square test was used for sex comparisons. Pearson’s correlation coefficient was used to estimate the strength of the correlation between each balance test and the PCFS. The absolute value of the correlation coefficient was interpreted as follows: 0.0 to 0.25 (no relationship), 0.25 to 0.50 (reasonable relationship), 0.50 to 0.75 (moderate to good relationship), and greater than 0.75 (very good to excellent relationship) [28]. Multivariable linear regression was used to determine whether the independent variables (Tinetti, FRT, MBT, BBS, and TUG) could predict PCFS. The balance test variables were entered into the model using the backward stepwise method for selection procedure, based on the significant increase in the coefficient of multiple determination (*R*^2^). The significance level was set at 5%.

## 3. Results

Sample characteristics are shown in Table 1, including sociodemographic and anthropometric data (without significant differences between groups; *p* > 0.05 for all cases), pre-existing comorbidities, and self-reported current symptoms. The time since COVID-19 infection in the post-COVID-19 group was at least one month (median and interquartile range: 47, 43–58 days).

**Figure 1:**
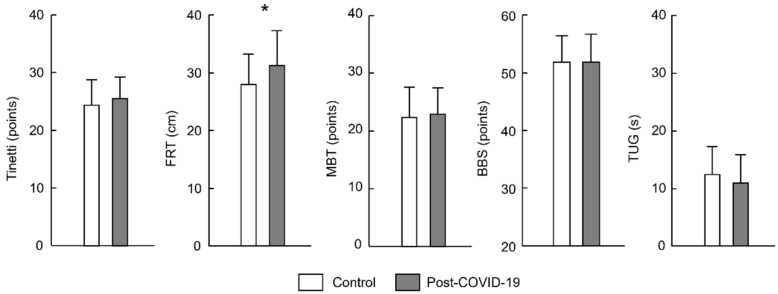
Mean (± standard deviation) of Tinetti, Functional Reach Test (FRT), Mini-BESTest (MBT), Berg Balance Scale (BBS), and Timed Up and Go (TUG) for each group, control (white color), and post-COVID-19 (gray color). Asterisk (*) indicates differences between groups (*p* < 0.05; n = 30 per group).

Figure 1 shows the results obtained in the groups for the balance tests applied. There were no significant differences between groups (control vs. post-COVID-19; Figure 1) for BBS (mean ± standard deviation; 49.2 ± 7.8 vs. 49.3 ± 8.3 points, *p* = 0.962); TUG (12.5 ± 4.9 vs. 11.0 ± 5.1 s, *p* = 0.262); Tinetti (24.4 ± 4.8 vs. 25.6 ± 3.8 points, *p* = 0.310); and MBT (22.5 ± 5.3 vs. 22.9 ± 4.7 points, *p* = 0.722). However, for the FRT, there was a significant difference (*p* = 0.046) between the post-COVID-19 group (31.3 ± 6.5 cm) and the control group (28.0 ± 5.7 cm).

**Figure 2:**
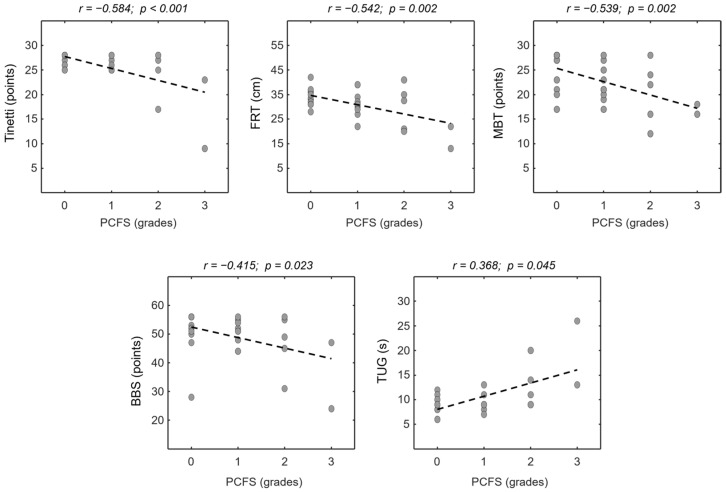
Relationships of PCFS grade with Tinetti, Functional Reach Test (FRT), Mini-BESTest (MB), Berg Balance Scale (BBS), and Timed Up and Go (TUG; post-COVID-19 group, n = 30). Pearson’s correlation coefficients were shown, and regression line was drawn for clarity.

Table 2 provides the assessment of functional status using the PCFS in the post-COVID-19 group. The results of the correlations are shown in Figure 2. All balance variables showed significant and moderate correlations with the PCFS as follows: FRT (r= −0.542 and *p* = 0.002); TUG (r = 0.368 and *p* = 0.045); BBS (r= −0.415 and *p* = 0.023); Tinetti (r= −0.584 and *p* < 0.001); and MBT (r= −0.539 and *p* = 0.002).

Regarding multivariable regression, Tinetti was the best independent (explanatory) variable that predicts the functional status in PCFS (adjusted *R*^2^ value of 0.476). Moreover, sex (with females as the reference group) also showed a significant association, indicating that being female is associated with a higher PCFS compared to males. All other balance variables were removed from the model since they reduce *R*^2^ by the smallest increment at each step (Table 3).

## 4. Discussion

This study aimed to investigate the effect of post-COVID-19 conditions on postural balance and its potential impact on functional status, as quantified by the PCFS scale, in non-hospitalized patients. We analyzed the correlations between different balance assessment tools and the PCFS scale to verify whether two or more variables of balance performance should be used to predict functional decline in post-COVID-19 individuals. Our main findings revealed the following: (i) similar postural balance between the control and post-COVID-19 groups for the BBS, TUG, Tinetti, and MBT, except for the FRT; (ii) moderate correlations between balance tests and the PCFS of the post-COVID-19 group; and (iii) the Tinetti test appears to play an important role in explaining functional status as assessed by the PCFS scale. These results may contribute to better assessments and interventions in the context of neuromuscular rehabilitation in post-COVID-19 individuals.

No significant differences in postural balance were found between the groups for most of the balance tests used. This can be attributed to the finding that COVID-19 patients generally do not have a high risk of falling in clinical tests assessing balance performance, as confirmed by previous studies. Regardless of the post-COVID-19 phase (acute or long), studies have shown a low fall risk for post-COVID-19 patients, although there are differences in clinical tests between patients and a control group [9,10,13]. For example, two studies showed post-COVID-19 patients with a time ≤10 s on the TUG test [9,10], indicating a low risk of falls [14,15]. Moreover, post-COVID-19 patients with a long time were also in the low levels for fall risk when considering the BBS and Tinetti scales [13]. In the present study, marginal differences in postural balance between groups may also be explained, at least in part, by the fact that the post-COVID-19 group showed marginal changes in motor function (23 out of 30 patients had a degree between zero and one according to PCFS—Table 2). This is supported by previous studies showing that balance changes are more pronounced in COVID-19 patients with a severe form of the disease in the acute phase [9,29]. This was likely due to vaccination, as COVID-19 vaccines are effective in controlling the pandemic where symptoms are marginal or absent in vaccinated individuals who become infected with new variants [30]. Unlike the other balance assessment tests, the FRT differed between groups (Figure 1). However, both groups had a reach greater than 17 cm, indicating low frailty and fall risk in patients [16,18]. Thus, on average, post-COVID-19 individuals who were not hospitalized and vaccinated appear to have marginal changes in postural balance.

Correlation analysis showed that the balance tests had a moderate and significant correlation with the PCFS. The correlations showed that greater functional impairment, estimated by the PCFS, was associated with the following: (i) smaller displacement in the FRT; (ii) longer execution time on the TUG test; and (iii) lower scores on the BBS, TINETTI, and MBT (Figure 2). Thus, individuals in the post-COVID-19 group who had mild functional limitations identified by the PCFS appeared to have changes in postural balance. Our findings may complement the study of Machado et al. (2021) [5], which, although showing the relationship of numerous symptoms with PCFS, suggests the inclusion of other measures often associated with the individual’s functional status, such as postural balance. Therefore, the PCFS appears to include deficits in postural balance in addition to other functional outcomes.

Among the balance assessment tests, the Tinetti test appears to play an important role in determining post-COVID-19 functional status as assessed by the PCFS. Our results showed that the Tinetti test was the only balance variable that improved the PCFS prediction model (adjusted *R^2^* value of 0.318, *p* < 0.001; Table 3). This appears to have implications for the assessment and intervention of motor rehabilitation in the post-COVID-19 context. The BBS and TUG tests are useful in assessing post-COVID-19 postural balance and functional capacity, respectively [21]. However, among the postural balance assessment tools used in the present study, postural balance assessed by the Tinetti test seems to be the most important clinical parameter predicting post-COVID-19 functional status by PCFS (Table 3). This finding suggests that the Tinetti test could be a tool to better assess postural balance during the clinical course of COVID-19 and its impact on functional status.

The present study has several limitations. A first limitation could be related to the lack of pre-COVID-19 values, which are recommended to assess changes in functional outcomes according to the PCFS guideline [5,6]. In addition, hospitalized individuals with severe forms of the disease were not included in this study. Previous evidence suggests that individuals with severe COVID-19 infections have greater balance deficits compared to healthy individuals, which would likely lead to greater functional impairment [9,10]. Additionally, COVID-19 symptoms were self-reported, limiting the ability to link balance test outcomes with symptom intensity, a potential issue for future studies aiming to enhance assessment tools. Finally, the motor function of our control group, comprising individuals with physical and/or intellectual alterations but without a prior COVID-19 diagnosis, may have masked balance differences between the groups. In this regard, healthy controls in the current study may have revealed balance changes in our post-COVID-19 individuals, following previous studies [9,10,13]. Despite these limitations, our results suggest that the assessment of postural control should be included in the repertoire of rehabilitation protocols for these patients and contribute to the implementation of the PCFS, an easily applicable tool for monitoring functional status.

## Figures and Tables

**Table 1:** Sample characterization (n = 60).

	Post-COVID-19 Group (n = 30)	Control Group (n = 30)
**Sociodemographic data**		
Female/Male	20/10	19/11
Age (years)	46.13 ± 14.32	49.53 ± 18.34
**Anthropometric data**		
Body mass (kg)	72.44 ± 12.37	67.84 ± 13.32
Height (m)	1.61 ± 0.08	1.61 ± 0.09
BMI (kg/m^2^)	27.75 ± 4.32	26.62 ± 4.52
**Pre-existing comorbidities, n (%)**		
Hypertension	9 (30.00%)	9 (30.00%)
Diabetes Mellitus	4 (13.33%)	1 (3.33%)
Disk herniation	3 (10.00%)	0 (0.00%)
Bariatric surgery	1 (3.33%)	2 (6.67%)
Post-operative breast surgery	1 (3.33%)	1 (3.33%)
Arthrosis	1 (3.33%)	1 (3.33%)
Not reported	14 (46.67%)	9 (30.00%)
**Actual symptoms, n (%)**		
Anxiety	4 (13.33%)	0 (0.00%)
Depression	2 (6.67%)	0 (0.00%)
Headache	6 (20.00%)	4 (13.33%)
Joint pain	8 (26.67%)	10 (33.34%)
Back pain	5 (16.67%)	15 (50.00%)
Fatigue	5 (16.67%)	1 (3.33%)
Dyspnea	2 (6.67%)	0 (0.00%)
Memory loss	2 (6.67%)	0 (0.00%)

Values are shown as absolute for sex and mean (±SD). Abbreviations: COVID-19, Coronavirus Disease 2019; BMI, Body Mass Index.

**Table 2:** PCFS results on post-COVID-19 group (n = 30).

	Post-COVID-19 Group
**PCFS**	
Grade 0—No functional limitation	13 (43.34%)
Grade 1—Very light functional limitation	10 (33.33%)
Grade 2—Light functional limitation	5 (16.66%)
Grade 3—Moderate functional limitation	2 (6.66)
Grade 4—Severe functional limitation	0 (0.00%)

The values shown are absolute and percentiles.

**Table 3:** Linear regression model for predicting the PCFS scale (n = 30).

Variables	*R* ^2^	Adjusted *R*^2^	Unstandardized Β	Standard Error	Standardized β	t	*p*
Model #1	0.590	0.434					
Sex			−0.946	0.360	−0.484	−2.626	0.016
Age (years)			0.003	0.015	0.043	0.192	0.849
BMI (kg/m^2^)			0.023	0.034	0.107	0.681	0.503
Tinetti			−0.132	0.068	−0.544	−1.949	0.065
FRT			−0.020	0.035	−0.139	−0.562	0.580
MBT			−0.012	0.063	−0.060	−0.191	0.850
Berg			0.050	0.061	0.441	0.811	0.427
TUG			0.084	0.088	0.459	0.959	0.348
Model #2	0.589	0.458					
Sex			−0.981	0.301	−0.502	−3.265	0.004
Age (years)			0.004	0.013	0.065	0.334	0.741
BMI (kg/m^2^)			0.022	0.033	0.102	0.673	0.508
Tinetti			−0.134	0.065	−0.555	−2.077	0.050
FRT			−0.022	0.032	−0.156	−0.688	0.498
Berg			0.043	0.049	0.380	0.881	0.388
TUG			0.074	0.070	0.406	1.061	0.300
Model #3	0.587	0.479					
Sex			−0.957	0.286	−0.490	−3.349	0.003
BMI (kg/m^2^)			0.024	0.032	0.111	0.757	0.457
Tinetti			−0.134	0.063	−0.555	−2.117	0.045
FRT			−0.026	0.030	−0.183	−0.883	0.386
Berg			0.044	0.047	0.392	0.931	0.361
TUG			0.079	0.067	0.433	1.180	0.250
Model #4	0.577	0.489					
Sex			−0.962	0.283	−0.492	−3.399	0.002
Tinetti			−0.136	0.063	−0.561	−2.163	0.041
FRT			−0.031	0.028	−0.220	−1.104	0.280
Berg			0.053	0.046	0.472	1.167	0.255
TUG			0.093	0.064	0.507	1.448	0.160
Model #5	0.555	0.484					
Sex			−0.969	0.284	−0.496	−3.410	0.002
Tinetti			−0.176	0.052	−0.727	−3.412	0.002
Berg			0.057	0.046	0.503	1.241	0.226
TUG			0.100	0.064	0.543	1.550	0.134
Model #6	0.528	0.473					
Sex			−0.906	0.283	−0.464	−3.207	0.004
Tinetti			−0.139	0.042	−0.573	−3.274	0.003
TUG			0.031	0.034	0.171	0.932	0.360
Model #7	0.512	0.476					
Sex			−0.827	0.269	−0.423	−3.077	0.005
Tinetti			−0.163	0.033	−0.673	−4.895	<0.001

Abbreviations: BBE, Berg Balance Scale; BMI, Body Mass Index; MBT, Mini-BESTest; TUG, Timed Up and Go; FRT, Functional Reach Test.

## Data Availability

The original contributions presented in the study are included in the article; further inquiries can be directed to the corresponding author.
